# Generalized Simultaneous Localization and Mapping (G-SLAM) as unification framework for natural and artificial intelligences: towards reverse engineering the hippocampal/entorhinal system and principles of high-level cognition

**DOI:** 10.3389/fnsys.2022.787659

**Published:** 2022-09-30

**Authors:** Adam Safron, Ozan Çatal, Tim Verbelen

**Affiliations:** ^1^Center for Psychedelic and Consciousness Research, Johns Hopkins University School of Medicine, Baltimore, MD, United States; ^2^Cognitive Science Program, Indiana University, Bloomington, IN, United States; ^3^Institute for Advanced Consciousness Studies, Santa Monica, CA, United States; ^4^IDLab, Department of Information Technology, Ghent University—imec, Ghent, Belgium

**Keywords:** SLAM, free energy principle, active inference, hippocampal and entorhinal systems, hierarchical generative models, robotics, artificial intelligence

## Abstract

Simultaneous localization and mapping (SLAM) represents a fundamental problem for autonomous embodied systems, for which the hippocampal/entorhinal system (H/E-S) has been optimized over the course of evolution. We have developed a biologically-inspired SLAM architecture based on latent variable generative modeling within the Free Energy Principle and Active Inference (FEP-AI) framework, which affords flexible navigation and planning in mobile robots. We have primarily focused on attempting to reverse engineer H/E-S “design” properties, but here we consider ways in which SLAM principles from robotics may help us better understand nervous systems and emergent minds. After reviewing LatentSLAM and notable features of this control architecture, we consider how the H/E-S may realize these functional properties not only for physical navigation, but also with respect to high-level cognition understood as generalized simultaneous localization and mapping (G-SLAM). We focus on loop-closure, graph-relaxation, and node duplication as particularly impactful architectural features, suggesting these computational phenomena may contribute to understanding cognitive insight (as proto-causal-inference), accommodation (as integration into existing schemas), and assimilation (as category formation). All these operations can similarly be describable in terms of structure/category learning on multiple levels of abstraction. However, here we adopt an ecological rationality perspective, framing H/E-S functions as orchestrating SLAM processes within both concrete and abstract hypothesis spaces. In this navigation/search process, adaptive cognitive equilibration between assimilation and accommodation involves balancing tradeoffs between exploration and exploitation; this dynamic equilibrium may be near optimally realized in FEP-AI, wherein control systems governed by expected free energy objective functions naturally balance model simplicity and accuracy. With respect to structure learning, such a balance would involve constructing models and categories that are neither too inclusive nor exclusive. We propose these (generalized) SLAM phenomena may represent some of the most impactful sources of variation in cognition both within and between individuals, suggesting that modulators of H/E-S functioning may potentially illuminate their adaptive significances as fundamental cybernetic control parameters. Finally, we discuss how understanding H/E-S contributions to G-SLAM may provide a unifying framework for high-level cognition and its potential realization in artificial intelligences.

## Introduction

“*We take almost all the decisive steps in our lives as a result of slight inner adjustments of which we are barely conscious*.”

*—W.G. Sebald*.

“*Not all those who wander are lost*.”

*—J.R.R. Tolkien, The Riddle of Strider, The Fellowship of the Ring*.

“*We shall not cease from exploration*

*And the end of all our exploring*

*Will be to arrive where we started*

*And know the place for the first time*.”

*—T.S. Elliot, Little Gidding*.

Autonomous systems face a fundamental challenge of needing to understand where they are positioned as they move through the world. Towards this end, roboticists have extensively investigated solutions to the problem of simultaneous localization and mapping (SLAM), whereby systems must infer both a map of their surroundings and their relative locations as they navigate through space (Cadena et al., 2016). Considering that these same challenges face any freely moving cybernetic system, natural selection has similarly exerted extensive teleonomical (i.e., illusory purposefulness) optimization in this direction (Dennett, 2017; Safron, 2019b), so generating mechanisms for enabling wayfinding and situating organisms within environments where they engage in multiple kinds of adaptive foraging. Perhaps the most sophisticated of all biological SLAM mechanisms is the hippocampal-entorhinal system (H/E-S), whereby vertebrates become capable of both remembering where they have been, inferring where they are, and shaping where they are likely to go next.

Here, we argue that the development of the H/E-S represented a major transition in evolution, so enabling the emergence of teleology (i.e., actual goal-directedness) of various forms (Safron, 2021b), ranging from governance by expected action-outcome associations to explicitly represented and reflexively modellable causal sequences involving extended self-processes. We focus on the implications of SLAM capacities *via* the H/E-S, and of evidence that this functionality may have been repurposed for intelligent behavior and cognition in seemingly non-spatial domains. We propose that all cognition and goal-oriented behavior (broadly construed to include mental actions) is based on navigation through spatialized (re-)representations, ranging from modeling abstract task-structures to temporal sequences, and perhaps even sophisticated motor control *via* SLAM with respect to body maps. Indeed, we would go as far as to suggest that the ubiquity of implicit and explicit spatial metaphors in language strongly points to a perspective in which cognition is centered on the localization and mapping of phenomena within both concrete and abstract feature spaces (Lakoff and Johnson, 1999; Bergen, 2012; Tversky, 2019).

In these ways, we believe Generalized Simultaneous Localization and Mapping (G-SLAM) may provide enactive groundings for cognitive science within the principles of ecological rationality (Todd and Gigerenzer, 2012). That is, we adopt a perspective in which cognition is traced back to its ultimate origins, wherein rationality is understood in terms of adaptations for shaping animal behavior in ways that further evolutionary fitness. Such ecological and ethological connections further provide bridges to optimal foraging theory and (generalized) search processes as ways of understanding cognition as a kind of covert behavior (Hills et al., 2013). While somewhat similar models of intelligence have been proposed (Hawkins, 2021), we suggest these other views may be somewhat misleading in neglecting to account for the central role of the H/E-S for realizing G-SLAM. In addition to providing an accurate viewpoint that grounds cognition in its cybernetic function as shaped over the course of evolution and development, G-SLAM will further allow rich cross-fertilization of insights between cognitive science and artificial intelligence. Given the particular functionalities enabled by the H/E-S, we propose this reverse-engineering project ought to be the central focus of cognitive science and machine learning, potentially constituting the most viable path forward towards realizing AI with advanced capacities for reasoning and planning (Bengio, 2017).

A thorough discussion of these issues is beyond the scope of a single manuscript. However, below we attempt to provide an overview of why we believe the G-SLAM perspective may provide a unification framework for cognitive science. First (in Section “LatentSLAM, a bio-inspired SLAM algorithm”), we review our work on biologically-inspired SLAM architectures for robotics. Then, we consider features of the H/E-S, including its functionality for localization and mapping in both physical and abstract domains. Finally, we discuss correspondences between features of SLAM and core aspects of cognitive functioning. We hope to explain how common principles may apply not only to the fundamental task of finding one’s way to desired locations in physical space, but for thought as navigation through abstract spaces. While much of what follows will necessarily be under-detailed and speculative, in subsequent publications, we (and hopefully others) will explore these issues in greater detail as we attempt to explain fundamental principles in neuroscience and artificial intelligence, while simultaneously seeking synergistic understanding by establishing conceptual mappings between these domains (Hassabis et al., 2017).

In the following section, we provide a high-level overview of LatentSLAM, which is also treated in greater detail in (Çatal et al., 2021a, b). While we believe many of these technical details may be relevant for explaining fundamental aspects of high-level cognition, a more qualitative understanding of this content should be sufficient for considering the conceptual mappings we (begin to) explore in this manuscript (Table 1). Section “The Hippocampal/Entorhinal System (H/E-S)” then summarizes current views on the H/E-S and its functioning in relation to spatial modeling and cognition more generally. Finally, Section “G(eneralized-)SLAM as core cognitive process” draws parallels between understanding in machines (using LatentSLAM) and humans (considering the H/E-S) and propose G-SLAM as a unification framework for cognitive science and artificial intelligence.

**Table 1:** Potential correspondences between LatentSLAM, cognitive psychological, and bio-computational phenomena.

**LatentSLAM**	**Cognitive-psychological processes**	**Bio-computational processes**
Mapping/graphing:	Inferring dimensions of feature spaces and relative locations of phenomena based on observations	Relations between hippocampal place cells for particular locations combined with entorhinal grid cells for multi-scale metric-affordance information
Localization:	Positioning specific phenomena (including the mapping and localizing system itself) within inferred feature spaces	Conjunction of hippocampal/entorhinal place/grid cells for positioning specific events within maps/graphs
Sensor and actuator uncertainty:	Perceptual (including mnemonic and imaginative) ambiguity	Body and world states are indirectly inferred based on partial information from noisy signaling systems
Views:	Visuospatial perception (as a function of actions)	Information from ventral and dorsal visual streams (and other modalities) organized according to egocentric perspectival reference frames (*via* posterior midline structures)
Proprioceptive poses:	Somatospatial perception (as a function of actions)	Frontal-parietal hierarchies over the somatomotor strip, with modeling/control potentially enhanced *via* explicit mapping of lateral parietal body schemas by other systems (e.g., midline structures coupling with the H/E-S)
Experience-map:	Structuring of episodic memory and imagination both informed by and informing visuospatial and somatospatial modalities	Transitions between hippocampal place fields entailing spatiotemporal trajectories for organisms (potentially including trajectories for important effector/sensor systems such as eyes and hands), both entrained by and entraining largescale cortical attracting states
Spatial landmark graphs:	Consciously-accessible representations of (salience-biased) spatial relations, potentially constituting our sense of space; semantic content of graph is based on actions and corresponding sensations as paths are traversed across/through these nodes	Hippocampal place fields as chained attractors, mutually entrained with cortex to orchestrate attracting states for population activity along reduced-dimensionality manifolds for both overt and covert action-perception cycles at and between these locations
Hierarchical generative model:	The processes by which a coherent stream of experience is generated and remembered with respect to both action and perception	A functional and algorithmic understanding of the brain as a hybrid machine learning architecture for predictive control of an embodied-environmentally-embedded agent
Fisher information metric:	The amount of information gained when traveling along a trajectory given a probabilistic generative model, wherein autonomous functioning is realized by minimizing discrepancies between predicted goal and present estimated states (*via* active inference); with respect to structure learning, the amount of “cognitive work” required to make sense of a domain	The amount of neural activity that must be expended to achieve adaptive cybernetic functioning in a given context, including with respect to constructing and refining world models entailed by patterns of effective connectivity
Accumulation of map uncertainty:	Deviations between models and that which is represented due to uncertainty with respect to cognition and latent world states	Deviations between likely patterns of neuronal attractor dynamics and their ability to orchestrate either overt or covert action-perception cycles (i.e., behaving or imagining) for autonomous functioning; cybernetic (and potentially thermodynamic) entropy for nervous systems
Loop-closures:	Events in which a familiar location in feature space is encountered with high confidence	High degrees of converging mutually consistent activity from the H/E-S and non-H/E systems
Graph-relaxation:	Assimilation of novel information into existing schemas *via* iterated distribution of updates across interconnected cognitive structures	Updating connectivity patterns to influence relative positioning of hippocampal place fields, potentially accompanied by largescale reductions in Hopfield energy
Node creation:	Accommodation of novel information *via* altering the structure of cognitive maps/graphs, potentially resulting in major updates to internal working (world) models with novel concepts	Creation of new place fields, involving various forms of (potentially neuromodulator-dependent) hippocampal plasticity, and/or establishment of new prefrontal attractors (i.e., patterns of canalized striatal-cortical loops)
Navigation:	Setting destinations in generalized space, which function as sources of prediction-error to be minimized through active inference; this may apply to the organism as a whole moving through (generalized) space, or to trajectories for parts of a system for which specific intentional control is warranted (e.g., directed ocular foveations or grasping/pointing movements), including with respect to spaces of a conceptual variety (e.g., spatialized time)	Predictive sweeps of activity across place fields from hippocampal maps (cf. successor representations), which can orchestrate largescale cortical attracting states (cf. equilibrium points) and thereby drive both system-internal self-organization (i.e., perceptual inference, imagination, and learning) and overt enaction, which in turn creates new sources of information to shape subsequent H/E-S dynamics

Please note, these cross-domain mappings are neither meant to be exhaustive nor definitive, but are instead intended to point in the direction of what a G-SLAM perspective might look like if more fully developed.

We realize that this may be a challenging manuscript for many readers, with some portions focused on describing a robotics perspective, and other portions focused on cognitive/systems neuroscience. Indeed, this article emerged from an ongoing collaboration between roboticists and a cognitive/systems neuroscientist, which has been both rewarding and challenging in ways that demonstrate why this kind of interdisciplinary work is both desirable and difficult. One of our primary goals for this manuscript is to provide a rough-but-useful conceptual scaffolding (i.e., an initial partial map) for those who would attempt such cross-domain research. In this way, interested readers ought not be overly concerned if some of the content is found to be excessively technical relative to their particular background. However, we believe readers who follow through with exploring these suggested mappings (which we only begin to characterize) may be richly rewarded for those efforts.

In brief, G-SLAM can be summarized as follows:

1. It is increasingly recognized that the H/E-S may be the key to understanding high-level cognition.
2. Within the field of robotics, the H/E-S has been identified as having been shaped by evolution for the problem of simultaneous localization and mapping (SLAM) for foraging animals, and where these capacities appear to have been repurposed for navigating through other seemingly non-spatial domains.
3. We believe it would be fruitful to explicitly think of the core functionalities of SLAM systems and test whether these are not just reflected in the functioning of the H/E-S with respect to physical navigation, but with respect to other high-level cognitive processes as well.

If the H/E-S is the kind of gateway to high-level cognition that it is increasingly suggested to be (Evans and Burgess, 2020; George et al., 2021; McNamee et al., 2021), and if it can be well-modeled as having been selected for SLAM functionalities that were later repurposed, then we believe the difficulty of exploring the following material will more than repay the effort of attempting to make the journey. We also ask readers to note places where spatial language can be found, only some of which was intentional. Indeed, we take such linguistic spatializations as supporting evidence for the G-SLAM perspective, which perhaps may be overlooked by virtue of its very ubiquity (cf. fish not noticing water). This is not to say that all spatial cognition points to a SLAM perspective. Yet we believe such spatial mappings are notable in affording opportunities for localization and mapping with respect to such domains. We leave it up to the discernment of our readers to assess how far one can go with following such paths through conceptual spaces, which may not only provide new perspectives on familiar territories on minds, but may even make inroads into discovering how we may follow similar paths to the destination of creating artificial systems with capacities that were formerly considered to be uniquely human.

## LatentSLAM, A Bio-Inspired SLAM Algorithm

Simultaneous localization and mapping (SLAM) has been a long standing challenge in the robotics community (Cadena et al., 2016). For autonomous functioning, a robot must try to map its environment whilst trying to localize itself in the map it is simultaneously constructing (i.e., SLAM). This setup creates a kind of “chicken and egg” problem in that a well-developed map is required for precise localization, but accurate location estimation is also required for knowing how to develop the map by which locality is estimated. This challenge is rendered even more difficult in that not only must the system deal with the seemingly ill-posed problem just described, but the inherent ambiguity of the environment is made even more difficult by sources of uncertainty from sensors and actuators. A fundamental challenge (and opportunity) with localizing and mapping is the detection of loop-closures: i.e., knowing when the robot re-encounters a location it has already visited. The challenge is due to the circular inference problems just described, and the opportunity is due to the particularly valuable occasion for updating afforded by the system having a reliable reference point in space. Such loop-closures have a further functional significance in allowing experiences to be bound together into a unified representational system where updates can be propagated in a mutually-constrained wholistic fashion, so providing a basis for the rapid and flexible construction and refinement of knowledge structures in the form of cognitive schemas that have both graph-like and map-like properties. With further experience, these schemata can then be transferred to the neocortex in the form of more stable adaptive action and thought tendencies, so forming a powerful hybrid architecture for instantiating robust causal world models (Hafner et al., 2020; Safron, 2021b).

**Figure 1:**
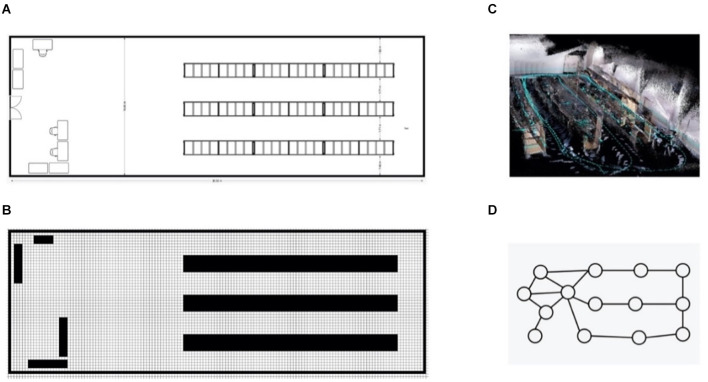
An overview of different map types, show-casing our robotics lab. Panel **(A)** gives an exact metric view of the room as drawn by an architect. Panel **(B)** shows the same map as a 2D grid map, to create this map from panel **(A)** the map was rasterized and untraversable terrain was filled into the granularity of a single raster cell. Pabel **(C)** shows the same room as an x, y, z mapping of red/green/blue values extracted from a RGBD camera. This 3D grid map was generated by moving the camera through the physical lab. Finally, panel **(D)** shows the lab as a sparse graph.

SLAM has traditionally been tackled by Bayesian integration of sensor information within a metric map, typically expressed in terms of absolute distances and angles. In previous work, this amounted to keeping track of distances between the robot and various landmarks in the environment. Distance measurements were typically combined through Bayesian filtering, a principled way of combining heterogenous information sources through Bayesian inference. Modern successful metric SLAM solutions, however, combine lidar scans with the robots internal odometry estimate through Kalman filtering (Kalman and Bucy, 1961) into 2D or 3D occupancy grid maps (Mur-Artal et al., 2015; Hess et al., 2016). These occupancy maps (Figures 1B,C) keep track of locations of objects in the environment by rasterizing space and then marking certain grid locations as inaccessible—due to being occupied with physical obstructions—so creating a map that resembles what an architect would create to diagram a room (Figure 1A).

Variations on this scheme are popular and differ wildly, either substituting the integration algorithm or the type of metric map. A metric map is akin to a Cartesian grid with regular spacings. However, such spatial maps do not speak to the object identities within the space of interest, nor the particular relations between those objects. Thus, one of the downsides of using metric maps is that by extension all robotic reasoning must also happen on a metric level, any semantic information (i.e., the meaning of a certain cluster of grid-cell activations) needs to be added in later. Further, such metric spaces represent an instance of deviating from natural designs, as hippocampal/entorhinal system (H/E-S) mappings are not independent of the objects contained within these spaces, but instead induce distortions (e.g., expansions and compressions) of spatial relations, which are also modulated as a function of the salience of these entities for the organism/agent (Bellmund et al., 2019; Boccara et al., 2019; Butler et al., 2019).

Popular approaches for such spatiotemporal modeling use particle filters or extended Kalman filters as Bayesian integration methods (Thrun et al., 2005). Kalman filters are notable in that they allow for estimation based on a precision-weighted combination of probabilistic data sources, so allowing for synergistic power in inference and updating, which is also theoretically optimal in making use of all available data (weighted by relative certainty). As will be discussed in greater detail below, such integration may be implemented in the H/E-S *via* convergent activation in regions supporting high degrees of recurrent processing, such as the CA3 subfield of the hippocampus. However, not only does the H/E-S promote integrative estimation, but also pattern separation/differentiation *via* other subregions such as CA1, so allowing for attractors to take the form of sparsely-connected graphs—cf. hybrid continuous/discrete architectures based on Forney factor graphs and agent-designs based on independently controllable factors (Friston et al., 2017b; Thomas et al., 2017, 2018). Below we will also describe how such graph-like representations not only help to solve problems in navigating through physical spaces, but may also form a basis for the kinds of high-level cognition sought after in the domain of neurosymbolic AI (Bengio, 2017).

We do not internally represent the world in a metric map. For instance, none of our senses can naturally give us an accurate distance measurement. Neither are we very effective in following a metric description of a path. Hence, it makes more sense for minds like ours (and potentially for artificial agents) to represent a map intuitively as a graph-like structure (Figure 1D), where subsequent graph-nodes could represent subsequent high-level parts of the environment e.g., a node could represent a part of the environment containing a door at a certain rough location. Map traversal then becomes equivalent to the potentially more intuitive problem of graph-traversal or navigating between meaningful landmarks. Trajectories can then be expressed in terms of consecutive semantically meaningful directions. For example, the metrical path “move forwards 2 meters, turn 90 degrees clockwise and continue for 2 meters” could become “after going through the door go right towards the table.” (Note: in vertebrate nervous systems, such forms of navigation could either be based on H/E-S graphs/maps, or occur *via* canalized striatocortical loops implicitly mapping states to actions, possibly with functional synergy, and also enhanced robustness (and thereby learnability) *via* degeneracy/redundancy.)

In LatentSLAM (Çatal et al., 2021a), we proposed a bio-inspired SLAM algorithm which tries to mimic this kind of intuitive mapping. With this architecture, we built topological, graph-based maps on top of a predictive model of the world, so allowing for separation of the low-level metric actions of the robot and high-level salient paths. Instead of using raw sensory data—or fixed features thereof (Milford et al., 2004)—directly as node representations, LatentSLAM learns compact state representations conditioned on the robot’s actions, which are then used as nodes. This latent representation gives rise to a probabilistic belief space that allows for Bayesian reasoning over environmental states. Graph nodes are formed from trajectories on manifolds formed by belief distributions. That is, rather than utilizing static maps, our agents navigate through space by moving between landmarks based on expectations of which state transitions are likely to be associated with those kinds of percepts. As an underlying foundation, LatentSLAM adopts the Free Energy Principle and Active Inference (FEP-AI) framework to unify perception (i.e., localization), learning (i.e., map building) and action (i.e., navigation) as a consequence of the agent optimizing one sole objective: minimizing its (expected) free energy (Friston, 2010; Friston et al., 2017a). As will be described in greater detail below, we believe this is an apt description of thinking as the unfolding of a stream of consciousness, with a variety of somatic states being generated in various combinations as the agent perceives and imagines itself moving through space and time.

### Representing the world in a graph

Graphs form a natural way of representing relations between various sources of information in a sparse and easily traversable manner. In LatentSLAM, such a structure is used to build a high-level map from agent experiences. This experience map contains nodes consisting of a *pose*, i.e., the agent’s proprioceptive information, and a *view* distilled from the sensory inputs. Together, the pose and view of an agent specify its unique experience: a different view in the same pose gives rise to a new experience; likewise, the same view from a different pose also constitutes a novel experience. Views generally lie on some learned compact manifold as a compressed version of one or more sensory inputs, integrated and updated through time. Links between experiences in the graph indicate possible transitions between one experience and another.

**Figure 2:**
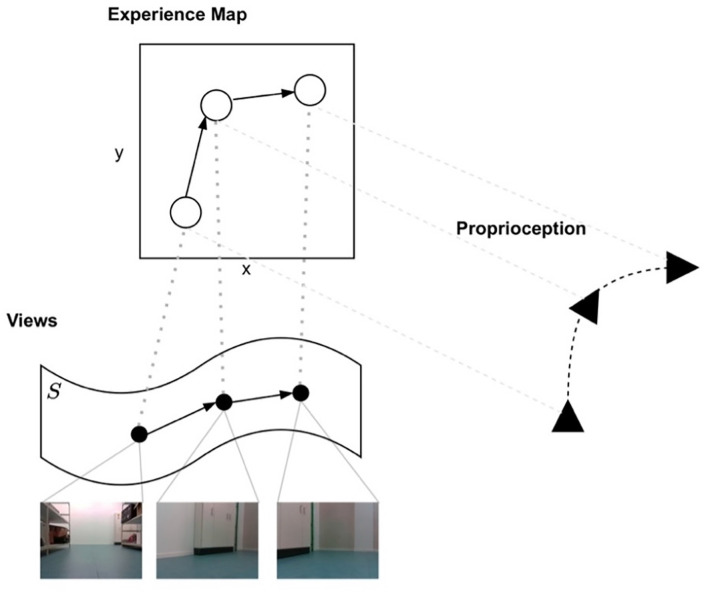
The formation of an experience-map out of views and proprioceptive poses. Sensory observations first need to be integrated into views to be compared to existing experiences from the graph. The shown graph is embedded in a Cartesian reference frame extracted from the proprioceptive information.

Figure 2 provides a visual overview of how poses and views combine into an integrated experience map. The pose information allows the agent to embed the graph relative to the geometrical layout of the environment. In this case, the embedding is done in 2D-Cartesian space as the example shows a ground based, velocity-controlled mobile robot. Embedding the graph in a reference frame correlated with environment characteristics organizes observations in ways that greatly enhance inferential power, since this avoids combinatorial explosions with respect to under-constrained hypothesis spaces. That is, a given sensory impression could correspond to an unbounded number of world states (e.g., something may be big and far away, or small and nearby), but coherent perspectival reference frames allow for likely causes to be inferred by mutually-constraining relevant contextual factors.

#### Experience map

The experience map (or graph) provides a high-level overview of the environment. Each node in the map represents a location in the physical world where the robot encountered some interesting or novel experience. These positions are encoded in poses in a spatial reference frame, e.g., a 2D-Cartesian space, whilst the experiences themselves are expressed as implicit representations of corresponding sensory observations. When view representations change according to distances to known landmarks, this setup resembles the approach described in the classical graphSLAM algorithm (Thrun and Montemerlo, 2006). Note that the seminal work on graph-based experience maps (Milford et al., 2004) also used an embedding of sensory observations into a lower dimensional space. However, in contrast to our approach, these mappings were deterministic and fixed for all observations.

The graph is embedded in, as opposed to being expressed in, a spatiotemporal reference frame, meaning that over time stored (or inferred) poses on the map are likely to exhibit deviations from their initial recorded values as they are progressively updated. Loop-closure events trigger a graph-relaxation phase wherein current graph nodes are re-positioned to take into account the unique opportunity accompanying the closing of the loop (i.e., the creation of a closed system of node linkages allowing for updating of the entire graph through energy minimization, accompanied by more confident location-estimation through experience-trajectory converging on known landmarks). This relaxation not only affords opportunities for map refinement, but it is also necessary due to the accumulation in pose errors from odometry drift. Wheel slippage, actuator encoder errors, and other similar effects amount to a continual increase in the uncertainty of the pose estimate. These sources of error/noise are part of what makes loop-closure such a hard problem in general. However, the loose embedding of pose information in the graph (combined with associated views) allows the map building to become robust to sensor and actuator drift, thereby maintaining a consistent map of the environment.

#### Views

LatentSLAM probabilistically learns views from sensory observations by incorporating the action trajectories from which they are generated, which differentiates our architecture from similar algorithms (Milford et al., 2004). The agent keeps track of a sample of the current belief distribution over states, which gets updated at each time-step into a new belief through variational inference. This sample constitutes either the current agent view, or a sensory-decoupled (or imagined) estimate of the environment from the latent space of the agent’s generative model. At each time-step, the agent inputs a conjunction of the current action, sample, and current observation into its generative model. This world model then generates a new state belief distribution based on the current state sample, which functions as a source of predictions for a predictive coding perceptual architecture. At training time, the generative model is tasked with predicting future observations based on previous recordings of trajectories through the environment.

#### Proprioception

An agent needs a principled way of keeping track of its estimated *pose* in the local environment. That is, an agent needs a coherent way to integrate changes in its local pose according to some local reference frame. In this form of proprioception, agents can estimate the effects of certain actions on local pose information relative to adjacent portions of its environment. This aspect of embodiment is essential in enabling consistent mapping and localization through challenging terrains.

In LatentSLAM this is handled through the low-level generative model on the one hand, and the pose continuous attractor network (CAN) on the other hand. The generative model allows for reasoning in terms of how actions affect views: i.e., it reduces the pose to an implicit part of the latent state representation. The CAN, however, leaves pose estimation as an explicit part of the greater LatentSLAM model. It integrates successive pose estimates through time in a multidimensional grid representing the agent in terms of internally measurable quantities. In the case of a ground-based mobile robot these quantities would be the expected difference in *x,y* pose and relative rotation of the robot over the z-axis. Hence, for a ground-based robot the CAN would be expressed as a 3D grid, that wraps around its edges. Sufficiently large displacements along the x-axis of this grid would teleport the pose estimate back to the negative bound of the same axis. This to accommodate for traversing spaces that are larger than the number of grid cells in the CAN. The pose estimate in the CAN is represented as an activation per grid cell, the value of which determines the amount of belief the model gives to the robot being in this exact relative pose. Multiple grid cell locations can be active at any given time, indicating varying beliefs over multiple hypotheses. The highest activated cell indicates the current most likely pose. Cell activity is generated in two ways: activity is added (or subtracted) to a cell through motion and the current proprioceptive translation thereof in terms of grid-cell entries; alternatively, activity may be modified through view-cell linkage. When a view is sufficiently different from others it gets added to the experience map together with the current most likely pose. This mechanism in turn allows experiences, when encountered, to add activation into the CAN at the stored pose estimate. This process can shift, and often correct, the internal pose estimate of the agent, allowing it to compensate for proprioceptive drift.

This conjunction of views and poses has notable parallels with neural representations decoded from respective lateral and medial entorhinal cortices (Wang C. et al., 2018), which constitute the predominant source of information for the hippocampal system (i.e., the experience map). It is also striking that the self-wrapping representational format for LatentSLAM poses/views recapitulates the repeated metric-spacing observed for entorhinal grid cells, whose location invariance may potentially provide a basis for knowledge-generalization and transitive inference across learning epochs and domains (Whittington et al., 2022). We believe that such correspondences between naturally and artificially “designed” systems constitutes strong evidence in support of a SLAM perspective for understanding the H/E-S.

### A hierarchical generative model

**Figure 3:**
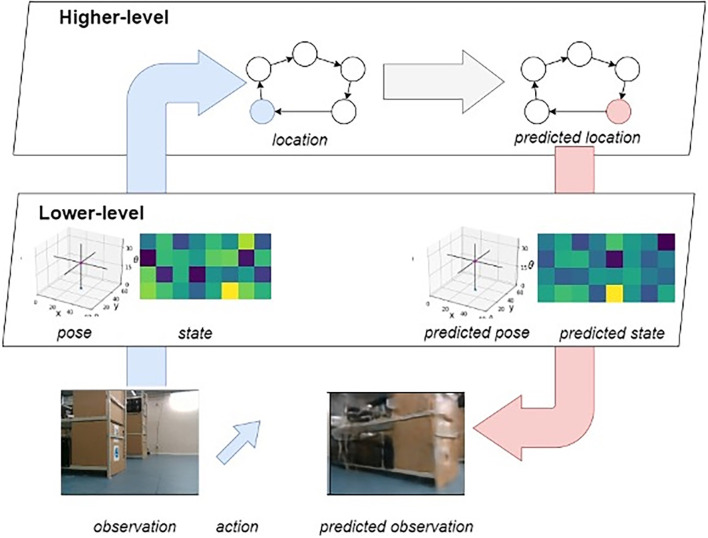
Overview of the hierarchical generative model. Highlighted in blue is the bottom-up sensory stream, and in pink the top-down prediction stream. As the agent moves about, it alternates between these two modes. On the one hand it will infer state information from the observations, and on the other hand it will predict future observations from inferred states.

The entirety of the LatentSLAM framework can be understood mathematically in terms of a hierarchical generative model (Figure 3; Çatal et al., 2021b).

There are two distinct levels of reasoning, each using their own generative model to explain the dynamics of the environment at the corresponding level of abstraction. As the generative models are stacked, the higher-level model takes the states from the lower level as observations, while the lower level observes the actual environment through the agents’ sensors. Each separate generative model can be seen mathematically as representing the joint probability $p(\tilde{o},\tilde{s},\tilde{a})=p(a_{0})p(s_{0})p(o_{0}|s_{0})\prod_{t=1}^{T}p(s_{t}|s_{t-1},a_{t-1})p(o_{t}|s_{t})$, with *o* relevant observations at each level; *s* state description, views or locations; and *a* possible actions at each level (either displacements in the environment or node transitions). These models only consider the generative process up until some future time horizon *T*. The exact instantiation of the joint probability and corresponding posterior distributions differ between each level of the hierarchy; interested readers are referred to Çatal et al. (2021b) for a more thorough description of this kind of model, and some extra details are provided in the “Appendix”.

Action and state inference, that is finding suitable instantiations of the posteriors $p(a_{t}|s_{t})$ and $p(s_{t}|s_{t-1},o_{t},a_{t-1})$ is achieved through Active Inference as understood in the context of the Free Energy Principle (FEP-AI; Friston et al., 2017a). In FEP-AI, intelligent agents are governed by predictive models that attempt to minimize variational free energy through updating of internal beliefs and modification of external states through enaction (hence, active inference). When implementing similar mechanisms in artificial agents such as robots, inference is amortized—cf. planning as inference *via* memorization of successful policies (Gershman and Goodman, 2014; Dasgupta et al., 2018)—through training variational auto-encoders (VAEs) with objective functionals that minimize (variational) free energy. The model consists of three neural networks, with each representing a conditioned probability distribution that outputs different multivariate Gaussian distributions based on differing inputs. These inputs can take the form of different sensor modalities such as lidar or camera; or they might be actions depending on the flow of information between neural networks.

State inference emerges naturally from the neural network architecture and training method. Active inference, however, leverages the trained network to create a set of imaginary trajectories from which optimal action sequences can be selected through expected free energy minimization. The model is trained on a free-energy objective functional, wherein it is tasked with minimizing Bayesian surprise—in the form of KL divergence—between prior and posterior estimates on the state. In this hierarchical generative model, there are two sources of information flowing in two directions at any given time. Sensory observations flow upwards from the real world through the lower-level pose-view model towards the higher-level mapping model. Predictions flow in the opposite direction, originating in the higher-level mapping model and flowing down into the environment through the predicted actions in the lower-level pose-view model.

#### Bottom-up sensory streams

The agent observes the world through sensors as it moves around the environment. At the lower-level of the generative model, the agent actively tries to predict future incoming sensory observations (Figure 3, blue arrow indicating informational flow). The agent actively abstracts away distractor elements in the observations as every observation gets encoded into a latent vector (i.e., views). As this encoding is generated from actions, observations and the previous latent state, the model considers the effects that history and actuation (or enaction) have on the environment. The abstracted view then gets fed into the higher-level mapping model which actively predicts the next experience from the previous one, taking into account the way the agent is presently traversing the experience graph and its current view.

#### Top-down prediction streams

At the same time, decisions flow down from the higher-level to the lower-level of the generative model (Figure 3, red arrow indicating informational flow). As a new navigational goal is set, the desired trajectory through the experience map is generated. Each node transition denotes one or more displacements in the real environment. While traversing the graph, the agent sets the views associated with the visited nodes as planning targets for the lower-level model. At the hierarchically higher level, the agent samples multiple state estimates from the current belief distribution over states and leverages the predictive capabilities of the generative model to envision possible outcomes up until some fixed planning horizon (Friston et al., 2021). From all these imagined future outcomes, the optimal one is selected after which the process repeats itself until the target view and pose are met. In turn the next node in the map trajectory is used to generate a new lower-level planning target.

### Creating the map

As mentioned earlier, once an agent encounters a sufficiently different experience, a new node is inserted in the experience map with the current view and pose. This process results in an ever-growing map of the environment as the agent explores the world. Hence, there needs to be a principled way to determine whether a view is new or is already known to the agent. As with many such problems, the solution presents itself in the form of a distance function in some well-defined mathematical space. A well-chosen distance function will allow the agent to not only build a consistent map of its environment but also account for loop-closure events.

#### Distance functions

Many SLAM algorithms use the Euclidian distance between poses to determine whether the current observation and pose are known in the map or represent some novel experience. However, due to the inherent drift in proprioception in many real-world scenarios, often this distance metric between poses and/or observations is not enough. Alternatives present themselves depending on the form of the probabilistic framework upon which the algorithm is based.

As described in Section “A hierarchical generative model”, LatentSLAM learns a latent state space manifold over sensory inputs (i.e., camera images). This enables the agent to not only evaluate Euclidian distances between poses, but also distances between two sensory inputs in the latent statistical manifold. To evaluate distances inside the manifold we need an appropriate distance measure. One notable candidate is the Fisher information metric (Costa et al., 2015), which represents informational differences between measurements. In our context, this means that two measurements are only encoded in different nodes of the experience map when there is sufficiently more information in one compared to the other. For example, moving in a long, white hallway with little texture will not yield a change in information in the latent manifold, hence this will be mapped on a single experience node. Only when a salient feature appears, for example a door, there will be enough sensory information to encode a new experience. In such a scenario however, methods building a metric map will likely fail as it is impossible to accurately track one’s position in a long, textureless hallway.

Note how the Fisher information metric is also related to the free energy minimization objective used for manifold learning. Concretely, if we take KL[x||x+δx] with x a probability distribution and x + δx a distribution close to x we get that if δx→0 then $KL[x||x+\delta x]\to \frac{1}{2}F(x)\;{\left(\delta x\right)}^{2}$. In other words, for infinitesimally small differences between distributions the KL divergence approaches the Fisher information metric (Kullback, 1959). This can be interpreted as integrating the agent’s Bayesian surprise over infinitesimal timesteps to measure the “information distance” traveled.

However, since the Fisher information metric and KL divergence do not have closed form solutions for many types of probability distributions, we use cosine similarity between the modes of the distribution as a numerical stable approximation function. Therefore, LatentSLAM evaluates *information* differences between experiences instead of differences in exact environmental observations.

#### Node creation and loop-closures

**Figure 4:**
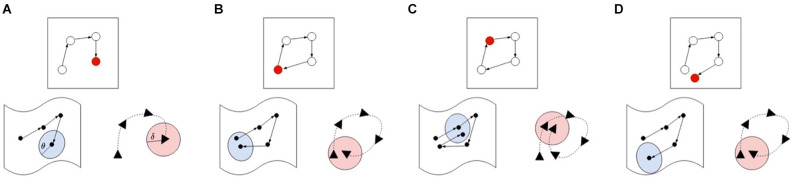
Different cases for illustrating the map updating procedure. For each case we show the map (top), pose (bottom right), and views (bottom left) in their own respective spaces. The current active map node is always indicated in red and the current pose or view value is the final one in the sequence. In case **(A)**, the agent encounters a new experience which is not within the threshold boundary of both the poses and views, so a new node is inserted into the map. Case **(B)** demonstrates a loop-closure event, where both the pose and view are within their respective thresholds, blue indicating the area pose information demarcated by its threshold θ, pink indicating the area covered by the view threshold. If both view and pose are within the threshold boundary (blue and pink) of the next node (case **C**), the estimate is shifted to the next node, skipping the current node in the graph. Finally, case **(D)** shows a matching pose without a matching view, requiring a new node insertion in the map.

When a salient landmark is identified, but the agent cannot find a single node in the graph which matches closely enough with the current view or pose, a new node must be inserted in the graph. Alternatively, if the current experience matches both on pose and view, a loop-closure is registered, but the agent leaves the map as is. In order to determine whether two experiences match, LatentSLAM uses a matching threshold *θ*. Both the pose and view of an experience is matched to experiences stored in the map. Figure 4 gives a visual overview of the various possible matching cases. If neither view nor pose match with any possible stored view or pose, a new experience is created and inserted into the map, as is shown in panel A. When the view and the pose both match, a loop-closure has occurred and the current experiences shifts to the stored experience, at which point a graph-relaxation phase is initiated. If the current observed experience matches with a stored experience further along the path, a relocation is required, and the estimate is shifted further along the path in the graph. Finally, if the current pose estimate matches a stored experiences pose, but does not find the corresponding matching view, a new node is inserted at the same location. This allows the agent to keep track of varying views of the same landmark throughout the map.

#### Graph-relaxation

As nodes are inserted throughout the graph, each new pose observation is subjected to sensor drift, leading to increasing errors for remembered poses. To address this issue, whenever a loop-closure event is encountered, graph-relaxation is applied to the experience graph. The algorithm treats every node in the graph as being connected with its neighbors as if suspended by weighted springs. The strength of each spring is related to the pose distances between the nodes. Then the algorithm reduces the total “energy content” of the graph by shifting the poses in such a way that the sum of the forces is minimized. This approach is similar to graph-relaxation in similar SLAM algorithms (Thrun et al., 2005; Thrun and Montemerlo, 2006). Graph-relaxation has the effect of morphing the shape of the pose embedding of the map to reflect the actual topology of the environment.

#### Setting the threshold

**Figure 5:**
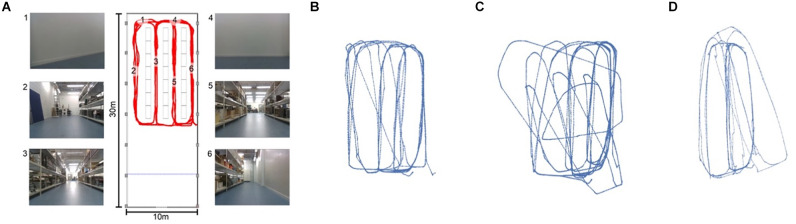
**(A)** Metric map of our lab environment, with some example camera views at the marked locations. The views at different locations (i.e., 3 and 5 or 1 and 4) appear very similar, making this a hard environment for visual SLAM. Panels **(B–D)** show three possible mappings of the trajectory shown in red in panel **(A)**. **(B)** With a well-tuned threshold θ^⋆^, our LatentSLAM algorithm recovers a topological map of the environment, clearly separating the four different aisles. **(C)** If the threshold is too stringent (*θ* ≪ θ^⋆^), loop-closure events are not detected, as every view is seen as unique, and the map becomes incorrect as proprioception errors (the main source of mapping errors) add up. **(D)** When the threshold is too relaxed (θ ≫ θ^⋆^), similar looking aisles are mapped onto each other due to false positive loop-closures.

Because the matching threshold has a significant impact on the shape and content of the map, it is one of the more important hyper parameters of LatentSLAM. For every environment there is an optimally tuned threshold parameter θ^⋆^. A matching threshold much lower than this optimal value will result in a mapping procedure with almost no loop-closure events. The map will contain every tiny permutation in views and poses as a separate node and will be insufficient in countering odometry drift. Conversely, if the threshold is set much higher than θ^⋆^, the mapping procedure will lump everything together in a small cluster of nodes. Figure 5 provides a visual example of the effects of the matching threshold on the resulting map.

### Navigation

Navigation is achieved through a dual process of first selecting nodes in the higher-level experience map, and then setting the node-views as targets for the active inference based lower-level action planner. In the first phase a path is generated through the graph connecting the current node and the target node. The final node is selected based on the visual reconstruction of the stored view. That is, the user of the system selects the view they want the system to have at a certain place. Once an experience trajectory is found, the agent can start acting in the environment. As each consecutive experience node is separated from its neighbors by a finite set of actions, a sequence of target views are extracted from the trajectory, forming the imaginary trajectory the agent may (approximately) bring about through overt enaction. The (active inference based) lower-level generative model is then capable of filling in further gaps between imagination and reality through additional planning.

At each step, the agent takes into account its current view and imagined trajectory up until the next target view. This imagination process leverages the learned intricacies and dynamics of the environment to compensate for the potential stochasticity in the interaction. Once a suitable trajectory is imagined at the higher-level, the agent enacts the first step of the trajectory, after which the lower-level planning process is repeated. These step-by-step transitions through the environment make the agent more robust against unexpected changes in the environment, which it might not have captured during model training.

Crucially, imagined trajectories are scored using a common objective functional of expected free energy, both on the higher level of proposed paths through the experience map/graph, as well as on the lower level of inferring actions capable of transitioning the agent between nodes (Çatal et al., 2021b). That is, trajectories are more likely to be selected if they bring the agent towards preferred outcomes and/or resolve uncertainty about the environment. Hence, action selection comprises a trade-off between instrumental value and epistemic value, which are naturally balanced according to a singular criterion of variational free energy. To provide an example in navigation, this tradeoff between the extrinsic value of realizing prior preferences and the intrinsic value of novel information could respectively manifest as either selecting a safer route *via* well-recognized landmarks or instead taking an unknown (but potentially shorter) path through a dark forest. Further, the discovery of such shortcut paths through space speaks to the kinds of flexible inference and learning that first motivated construals of the hippocampal system in terms of cognitive maps (Tolman, 1948), and in a G-SLAM context could be thought of as a way of understanding a core aspect of intelligence in the form of creative insight. And in the context of AI, such creative cognition may afford the creation of much sought after capacities for powerful inferences and one-shot learning in novel situations, which if realized could greatly enhance autonomous functioning.

### Limitations and future directions

There are several limitations with the current implementation of LatentSLAM. First, the experience graph is incapable of merging nodes with similar views and approximately similar poses into a single unified stochastic node. This in turn leads the algorithm to generate an increasing number of nodes for each pass through a single location. Second, the lower-level planning is limited to the sequence length encountered during training, and as such the model is incapable of imagining coherent outcomes beyond this time horizon. This brings us to a potentially substantial limitation of LatentSLAM, in that the lower-level generative model needs to be pre-trained on the types of observations it can encounter in the environment. That is, when the target views are unknown, imaginative planning may be required wherein agents visualize an assortment of potentially rewarding (counterfactual) action-outcome pairings. Going forward, we aim to alleviate these constraints by adapting the training procedure to accommodate online learning, allowing the agent to learn to imagine whilst exploring (Safron and Sheikhbahaee, 2021), which may be understood as a kind of deep tree search through policy space *via* Markov chain Monte Carlo sampling (Dohmatob et al., 2020; Friston et al., 2021), with potentially relevant insights obtainable from advances in Bayesian meta-reinforcement learning (Schmidhuber, 2020).

To extend the biological fidelity (and potential functional capacities) of our architecture, we intend on attempting to recapitulate particular empirical phenomena such as the specific conditions under which new place fields are introduced or pruned away in mammalian nervous systems. For example, the insertion of environmental barriers or encountering corridors leading to identical rooms may induce duplication of sensory views at different locations, which may speak to the phenomenon of place-field duplication—which in a LatentSLAM context would involve node creation (Lever et al., 2009; Spiers et al., 2015)—yet where these representations may also disappear with further learning. This kind of pruning of nodes—potentially involving “artificial sleep”—could be a valuable addition to latent SLAM’s functionality, and may potentially be understood as an instance of Bayesian model reduction with respect to structure learning (Friston et al., 2019), so providing another means by which capacities for creative insight (in terms of discovering more elegant models) may be realized in AI.

With respect to these particular phenomena involving challenging ambiguous situations, we may speculate that highly-similar-but-subtly-different pose/experience map combinations could represent instances associated with high levels of prediction-error generation due to a combination of highly precise priors and contradictory information. Speculatively, this could be understood as an example of “hard negative mining” from a contrastive learning perspective (Mazzaglia et al., 2022). As will be described in greater detail below, such highly surprising events may be similar to experiences of doorway or threshold crossing, and may trigger the establishment of event-boundaries *via* frame-resetting and spatial-retiling. Speculatively, the assignment of particular content to particular rooms in “memory palaces” could be understood as a necessary part of the art of remembering due to this phenomenon potentially interfering with semantic “chunking” (or coherent co-grounding). In attempting to apply LatentSLAM to cognition more generally, it could potentially be fruitful to look for generalizations of these phenomena with respect to seemingly non-spatial domains, such as with respect to creativity and insight learning problems in human and non-human animals.

Finally, and with further relevance to realizing capacities for imaginative planning and creative cognition, we will attempt to include phenomena such as sharp-wave ripples and forward/reverse replay across hippocampal place fields (Ambrose et al., 2016; de la Prida, 2020; Higgins et al., 2020; Igata et al., 2020), which have been suggested to form a means of efficient structural inference over cognitive graphs (Evans and Burgess, 2020). With respect to our goal-seeking agents, forward replay may potentially help to infer (and prioritize) imagined (goal-oriented) trajectories, and reverse replay may potentially help with: (a) back-chaining from goals; (b) increasing the robustness of entailed policies *via* regularization, and (speculatively), and (c) allowing for a punishment mechanism *via* inverted orderings with respect to spike-timing-dependent-plasticity. In these ways, not only may a G-SLAM approach allow for deeper understanding of aspects of biological functioning, but attempting to reverse engineer such properties in artificial systems may provide potentially major advances in the development of abiotic autonomous machines.

## The Hippocampal/Entorhinal System (H/E-S)

The hippocampal/entorhinal system (H/E-S) represents a major transition in evolution (Gray and McNaughton, 2003; Striedter, 2004), with homologs between avian and mammalian species suggesting its functionality becoming established at least 300 million years ago (Suryanarayana et al., 2020), with some of its origins potentially traceable to over 500 million years in the past with the Cambrian explosion (Feinberg and Mallatt, 2013), and potentially even earlier. It may be no overstatement to suggest that the H/E-S represents the core of autonomy and cognition in the vertebrate nervous system, with similar organizational principles enabling the potentially surprising degrees of intelligence exhibited by insects (Ai et al., 2019; Honkanen et al., 2019).

While their precise functional roles continue to be debated, the discovery of hippocampal place cells and entorhinal grid cells was a major advance in our understanding of how space is represented in the brain (O’Keefe and Nadel, 1978; Hafting et al., 2005). Similarly important was the discovery of head direction cells in rats, which were found to activate according to moment-to-moment changes in head direction (Sharp PE, 2001). Place cells have been modeled as representing a “predictive map” based on “successor representations” of likely state transitions for the organism (Stachenfeld et al., 2017), and grid cells have been understood as linking these graphs (or Markov chains) to particular events happening within a flexible (multi-level) metric tiling of space, so allowing for estimates of locations *via* path integration over trajectories. While we need not resolve the precise correspondences between these cell types here, there are intriguing developmental observations of place cells acquiring more mature functioning prior to grid cells, both of which likely depend on head-direction cells for their emergence (Canto et al., 2019; Mulders et al., 2021). In other contexts, place-specific cells have been found to index temporal sequence information, potentially functioning as “time cells” (Pastalkova et al., 2008), so providing a further means by which the H/E-S may provide foundations for coherent sense-making and adaptive behavior through the spatiotemporal organization of organismic information (Eichenbaum, 2014; Umbach et al., 2020).

In addition to place, time and grid cells, a variety of additional specialized cell types have been observed in the H/E-S. While it was previously assumed that these features represent innate inductive biases (Zador, 2019), increasing evidence suggests these specialized cell types may arise from experience-dependent plasticity, including models with similar architectural principles to the ones described here. In recent work from DeepMind (Uria et al., 2020), a recurrent system was used to predict sequences of visual inputs from (the latent space) of variational autoencoders. A natural mapping from egocentric information to an allocentric spatial reference frame was observed, including the induction of specialized units with response properties similar to head direction, place, band, landmark, boundary vector, and egocentric boundary cells. Similar results have been obtained with the Tolman-Eichenbaum machine (Whittington et al., 2020), including demonstrations of reliable cell remapping, so enabling transfer learning across episodes with the potential for the creative (re-)combination of ideas and inferential synergy. Other intriguing work on the emergence of specialized H/E-S functions through experience comes from work on “clone-structured cognitive graphs”, where various aspects of spatial maps are parsimoniously formed as efficient (and explanatory) representations of likely state transitions through the duplication and pruning of nodes in a dynamically-evolving sequence memory (George et al., 2021). While this evidence suggests a potentially substantial amount of experience-dependence in the emergence of the “zoo” of specialized neurons for spatiotemporal navigation, the development of these features still involve clear innate inductive biases (Zador, 2019). Specifically, specialized pathways ensure that the H/E-S receives neck-stretch-receptor information from the mamillary bodies and yaw/pitch/roll information from the vestibular apparatus (Papez, 1937; Wijesinghe et al., 2015), so providing bases for sensor-orientation with respect to head-direction and thereby the foundations of egocentric perspective.

### H/E-S as orchestrator of high-level cognition

While the association of the hippocampus with autobiographical and declarative memory is well-documented (MacKay, 2019), the H/E-S is increasingly being recognized as foundational for cybernetic functioning on multiple scales. A more thorough understanding of the principles governing the H/E-S and its interactions with the rest of the brain may allow us to understand how such sophisticated cognition and behavior is demonstrated by biological organisms (Todd and Gigerenzer, 2012). Even more, such knowledge may also allow us to find ways of reproducing these functionalities in artificial intelligences.

The hippocampus is usually described in terms of a “trisynaptic circuit” (Andersen, 1975), with multiple specialized subsystems that interact with functional synergy. The dentate gyrus is the primary input area to the hippocampus from entorhinal cortex, with densely packed cells for pattern separation, so allowing for multiple separable/orthogonal representations. Much of this information then feeds into CA3, characterized by highly recurrent circuits with tight loops for dynamic pattern completion. This information is then routed to CA1, characterized by sparse and stable representations, representing the primary output area of the hippocampus and interface with the rest of the brain. Taken together, the subfields of the hippocampal complex allow multiple sources of information to be not just independently stored in memory, but also creatively combined within and across experiences, so affording powerfully synergistic functionalities such as transfer learning and generalizable knowledge. Intriguingly, some evidence suggests that humans might be unique in exhibiting less pattern separation in their hippocampal subfields, potentially contributing to—and possibly being a function of—cognition involving high degrees of abstraction/invariance (Liashenko et al., 2020; Mok and Love, 2020; Quiroga, 2020).

Indeed, the functional properties enabled by the H/E-S represent the state of the art in machine learning for real world applications such as autonomous vehicles and artificial intelligences attempting to realize higher-order reasoning abilities (Ball et al., 2013; Bengio, 2017; Hassabis et al., 2017; Kaplan and Friston, 2018; Shang et al., 2019; Eppe et al., 2020; Greff et al., 2020; Parascandolo et al., 2020; Shamash et al., 2020; Friston et al., 2021). This is a bold claim for a system that might be describable as an association machine or spatial mapper, which when lesioned tends to leave much of higher-order intelligence intact. However, closer inspection of hippocampal patients reveals its essential contributions to complex reasoning, emotion, and general behavioral flexibility (MacKay, 2019). It should also be kept in mind that while someone might be able to maintain certain functions after losing a system in adulthood—as this functionality may become distributed throughout the rest of the brain with experience—the congenital absence of a working H/E-S might be a wholly different manner, potentially precluding the bootstrapping of any kind of sophisticated cognition or coherent world modeling whatsoever (Safron, 2021a). Further, principles of association may be surprisingly powerful if they are capable of representing specific relational structures as particular graphs/networks, which are increasingly being recognized as powerful learning and inferential systems (Gentner, 2010; Zhou et al., 2019; Crouse et al., 2020). Some have even suggested that the mapping abilities of the H/E-S may provide bases for a potential core functionalities associated with conscious processing in the form of “unlimited associative learning” (Birch et al., 2020), in which knowledge may be flexibly aggregated across experiences (Mack et al., 2018, 2020; Mok and Love, 2019)—cf. transfer and meta-learning (Wang J. X. et al., 2018; Dasgupta et al., 2019; Kirsch and Schmidhuber, 2020). The central role of the H/E-S for higher-order cognition is further understandable in light of the fact that many (and possibly most) aspects of intelligence can be described as search processes (Conant and Ashby, 1970; Hills et al., 2010), which might be even more clearly apparent if we think of the possibility of spatializing abstract domains such as complex feature spaces (Eichenbaum, 2015; Whittington et al., 2018), or even time (Howard, 2018; Gauthier et al., 2019).

The H/E-S represents both the developmental foundation and functional apex of the cortical hierarchy (Hawkins and Blakeslee, 2004; Barron et al., 2020). In predictive processing models of the brain—e.g., the variational autoencoder framework described here—observations not predicted at lower levels eventually reach the entorhinal cortex and hippocampus. We propose the H/E-S allows these high-level prediction-errors to be temporarily encoded and organized with spatiotemporal and abstract relational structure for informational synergy. Indeed, on a high-level of abstraction, the H/E-S can be considered to be a kind of Kalman variational autoencoder that combines heterogeneous forms of (precision-weighted) information for SLAM in generalized state/phase space (Fraccaro et al., 2017; Zhang et al., 2017). Alternatively framed, the cortical predictive hierarchy can be viewed as hierarchical Kalman filtering all the way up and all the way down (Friston, 2010). Along these lines, it is notable that the H/E-S itself may operate in a manner that reflects more general principles of cortical predictive processing. With canonical microcircuits for predictive coding, predictions are associated with deep pyramidal neurons and alpha/beta frequencies, and prediction-errors are associated with superficial pyramidal neurons and gamma frequencies (Bastos et al., 2012, 2020). Consistently with the H/E-S involving predictive processing, novel information (i.e., prediction errors) induce activation of superficial pyramidal neurons for entorhinal cortex, dentate, and CA3, and recollection (i.e., predictions) are associated with activations in deep pyramidal neurons for CA1 and entorhinal cortices (Maass et al., 2014). Also consistently with a predictive processing account, another study observed superficial place cells in CA1 responding (*via* a rate code) in cue poor-environments, and deep pyramidal neurons responding (*via* a phase code) in cue-rich environments, where we might respectively expect either prediction-errors or predictions to predominate (Sharif et al., 2020).

From a predictive coding perspective, the hippocampus is a strange kind of cortex, not only because of its particular cytoarchitectonic properties (e.g., 3 vs. 6 layers), but also because of its connectomic centrality. Some proposals have suggested that memory recall may arise from “fictive prediction errors” (Barron et al., 2020)—a perhaps somewhat counter-intuitive suggestion, in that the hippocampus is considered to be the top of the cortical heterarchy, and hence would be expected to only provide descending predictions—so providing a source of training signals for optimizing generative models of the world without sensory data, as well as affording stimulus-independent learning and imaginative planning. This is consistent with work from DeepMind in which the hippocampus is described as operating according to principles of “big loop recurrence”, where its outputs can be recirculated as inputs for offline learning and counterfactual processing (Koster et al., 2018). Indeed, the H/E-S may not only provide sources of predictions for the neocortex, but potentially prediction-errors for itself, possibly by parameterizing simulations from cortical generative models (Higgins et al., 2020). Further, recent evidence regarding episodic memory formation and retrieval suggests that interactions between cortex and the H/E-S may reflect the roles of various frequency bands in predictive coding, or “routing” (Griffiths B. J. et al., 2019; Bastos et al., 2020). In this work, neocortical alpha/beta (8–20 Hz) power decreases reliably correlated with subsequent hippocampal fast gamma (60–80 Hz), and hippocampal slow gamma (40–50 Hz) power, potentially indicative of a trading off between predictions and prediction errors. However, this is somewhat different than the standard predictive coding account attributed to the cortex more generally, in that gamma frequency involvement may support the aforementioned idea that hippocampal reactivation of memories involve “fictive prediction errors” (Barron et al., 2020), rather than a suppressive explaining away.

In contrast to other slow rhythms, hippocampal theta oscillations may indicate enhancement of observations *via* cross-frequency phase coupling (Canolty and Knight, 2010), potentially providing a basis for high-level action and attentional selection. Along these lines, the ability of theta-oscillations to select and orchestrate cortical ensembles at gamma frequencies may provide a role for the hippocampal system as a comparator, enabling contrasting between percepts, whether based on observations or imagination (Safron, 2021b). Opposite phase relations between CA1 and CA3 (Tingley and Buzsáki, 2018) are suggestive, potentially indicating both a kind of predictive coding within the hippocampal system, and possibly also instantiating and orchestrating the formation and contrasting of corresponding cortical ensembles as alternating phases of duty cycles for theta oscillations (Heusser et al., 2016; Kunz et al., 2019). Indeed, the entertainment of counterfactuals might not only depend on a cortical hierarchy of sufficient size to support an inner loop separable from immediate engagement with the sensorium (Buckner and Krienen, 2013), but also a working H/E-S to stabilize ensembles associated with novel (due to being non-actual) possibilities (Hassabis et al., 2007). In this way, in conjunction with the rest of the cortex, the H/E-S could be viewed as an energy-based self-supervised contrastive learner (Mazzaglia et al., 2022), which may enable a substantial amount of adaptive-autonomous behavior if (variational) free-energy/prediction-error is being minimized with respect to divergences between goals and present estimated states (Hafner et al., 2020; Safron, 2021b).

It has recently been suggested by researchers at Numenta (a biologically-inspired AI company) that the principles (and particular cellular adaptations such as grid cells) involved in H/E-S functioning—e.g., allocentric object modeling (Sabour et al., 2017; Kosiorek et al., 2019)—may be recapitulated throughout the entire neocortex (Hawkins et al., 2019). The idea that the H/E-S may represent a template for understanding the neocortex is not unreasonable, since while it is referred to as “subcortical”, it is technically composed of cortical tissue (Insausti et al., 2017). Along these lines, not only is the H/E-S topologically central as a “convergence divergence zone” (Damasio, 2012) and hub for “semantic pointers” (Blouw et al., 2016), but it is also primary from an evolutionary (as archaecortex/periallocortex) and developmental perspective.

Modeling based on object-centered reference frames may be a broader property of the neocortex (Hawkins, 2021). However, we believe that such coherent perspectives may depend on being able to conduct active inference and learning with sufficient degrees of independence from other modeling/control processes (Thomas et al., 2017, 2018). That is, we suggest that for emergent modules to have H/E-S properties, they must be able to achieve informational closure with sufficient rapidity that they can both independently inform and be informed by action-perception cycles with respect to particular effector-sensor systems. For example, the establishment of such independently controllable factors may be the case for large macrocolumns such as rodent whisker barrels, but potentially not for ocular dominance columns. To the extent that hippocampal and entorhinal cell-types are found more generally throughout the cortex (Long and Zhang, 2021), we suggest that it remains ambiguous as to whether this reflects G-SLAM constituting a common cortical algorithm, or whether such representations are induced over the course of development *via* integrative functioning involving the H/E-S.

### The H/E-S as sense-maker and value integrator/realizer

Switching between conceptual scenes involves ramping of hippocampal activity, followed by high-frequency signaling with the cortex as a new frame of sense making is established (de la Prida, 2020; Karimi Abadchi et al., 2020; O’Callaghan et al., 2021). Theoretically, these events (potentially accompanied by sharp wave ripples) would represent the formation of new grid/place tiling/mapping/graphing over a space/scope of relevance, but where sufficient functionality is carried over across remappings for integration of information across episodes. Functionally speaking, these frame-shifts could be understood in terms of Lévy flights with respect to generalized search, so allowing for more exploratory processing and creative solutions in the face of challenges (Hills et al., 2010; McNamee et al., 2021). That is, in contrast to searching *via* random walks that would tend to result in reliable exploitative mapping of simple domains, such discontinuous (and potentially fanciful) flights to remote areas of hypothesis/phase spaces would allow agents to both more efficiently explore complex domains and escape from local optima. Considering that the H/E-S may be understood as the highest (or most flexibly integrative) level of agent-level control processes, altering parameters/modulators relevant for this kind of more exploitative or exploratory (generalized) search could be some of the most significant sources on variation both between and within individuals and species (Safron, 2020c).

While the precise conditions for remapping are likely to vary based on multiple conditions, degree of overall prediction-error seems to be one reliable trigger, as in an experiment in which participants were cued to retrieve well-learned complex room images from memory and then presented with either identical or modified pictures (Bein et al., 2020); in this study, the number of changes caused CA1–CA3 connectivity to decrease (potentially indicating less intra-hippocampal recurrent activity) and CA1-entorhinal connectivity to increase. Consistently, another study found sensitivity to reward prediction errors with respect to the establishment of new event boundaries (Rouhani et al., 2020). Similar influences on the stability of mappings by more general salience is suggested by studies in which the H/E-S shows sensitivity to interactions with the amygdala and responses to fearful stimuli (Chen et al., 2019), as well as modulation of encoding based on attention/expectancy (Mack et al., 2018, 2020; Urgolites et al., 2020). The dividing of continuous unfoldings into discrete epochs provides another means by which abstract phenomena such as time may be conceptualized by the H/E-S (in addition to their spatialization, perhaps as a kind of multidimensional scaling onto lower dimensional manifolds that may be inspected either through fictive navigation or imaginative visual foraging (Ramachandran et al., 2016).

Notably, the H/E-S may not just be sensitive to reward, but it may also help to provide a major source of the prediction errors that drive phasic dopamine (Mannella et al., 2013; Ballard et al., 2019; Jang et al., 2019; Laubach et al., 2020), potentially involving internal contrasting between hippocampal subfields, and with overall prediction-error being further integrated *via* outputs to the subiculum (Tingley and Buzsáki, 2018; Canto et al., 2019). This may allow for the allostatic prioritization of goals with respect to not only cortical predictions from medial prefrontal cortices, but even homeostatic regulatory nuclei of the septum (Tingley and Buzsáki, 2018; Kunz et al., 2019; Livneh et al., 2020). The importance of the H/E-S for motivational states is also evidenced by its ability to influence the interoceptive components of emotions (Edwards-Duric et al., 2020), which may have a further (circular) causal significance in helping to drive counterfactual simulations, potentially understandable as affectively-canalized Markov chain Monte Carlo tree search through value space (Dohmatob et al., 2020; Hesp et al., 2020; Parascandolo et al., 2020; Safron, 2021b). In this way, not only would the H/E-S help implement SLAM processes with respect to both concrete and abstract cognition, but it may also help to explain how agent-level mental processes can enter causal streams leading to both mental simulations and overt enaction, so affording some of the varieties of “free will” worth having for autonomous systems (Safron, 2021b).

**Figure 6:**
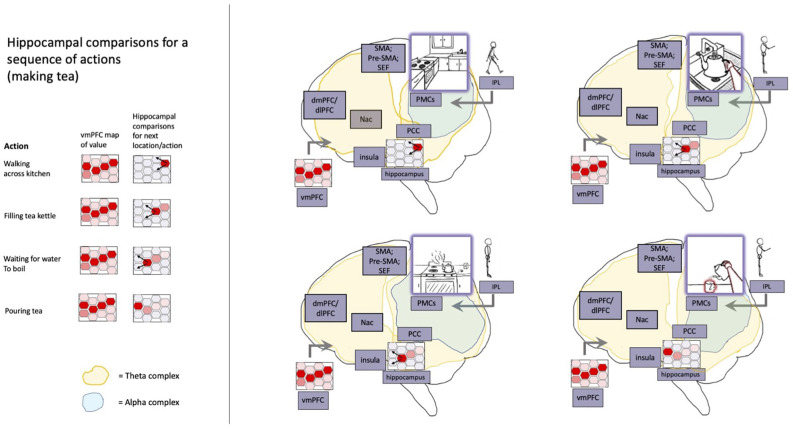
A model of hippocampally-orchestrated imaginative planning and action selection *via* generalized navigation. Goal-oriented action sequences are depicted with respect to relevant neural processes. The hippocampal system provides (a) organization of cortical attracting states into value-canalized spatiotemporal trajectories, (b) stabilization of ensembles *via* theta-mediated cross-frequency phase coupling, and (c) goal-oriented cognition and behavior *via* contrasting (not depicted) sensed and imagined states. Hippocampal trajectories are shaped according to whichever paths are expected to result in more positively valanced outcomes (cf. reward prediction errors). The expected value associated with navigating to different portions of (potentially abstract) space is informed *via* coupling with similarly spatiotemporally-organized value representations (red shaded hexagons) in vmPFC and associated systems. As chained patterns of activity progress across hippocampal place fields (red hexagons with variable degrees of shading), theta-synchronized frontal ensembles (yellow shading spreading towards the front of the brain) help to generate (*via* cross-frequency phase coupling) ensembles for directing attention, working memory, and overt enaction. Sensory updating of posterior cortices occurs at alpha frequencies (blue shading), so providing a basis for conscious perception and imagination. With respect to these integrated estimates of sensory states, hippocampal coupling at theta frequencies (yellow shading spreading towards the back of the brain) provides a basis for (a) episodic memory and replay, (b) novel imaginings, and (c) adjustment of neuronal activity selection *via* orchestrated contrasting between cortical ensembles. Abbreviations: nAC, nucleus accumbens; vmPFC, ventromedial prefrontal cortex; dmPFC, dorsomedial prefrontal cortex; SMA, supplementary motor area; Pre-SMA, presupplementary motor area; SEF, supplementary eye fields; PCC, posterior cingulate cortex; PMCs, posterior medial cortices; IPL, inferior parietal lobule. Reprinted with permission from Safron (2021b).

Some evidence for this affective influencing of H/E-S dynamics may potentially be found in studies of increased inter-hemispheric phase coupling (delta range coherence) during treadmill running periods (Furtunato et al., 2020), potentially corresponding to periods of increased driving by biophysical signals indicating organismic salience. Crucially, sources of H/E-S “reward” may not just take the form of the aforementioned extrinsic value of goal realization, but may also be driven by the intrinsic value of novel information, for the hippocampus could provide a natural integrator of prediction-error as top of the cortical hierarchy (Hawkins and Blakeslee, 2004; Mannella et al., 2013; Fonken et al., 2020). While the hippocampus and ventromedial prefrontal cortex may usually work together in estimating expected value (or opportunities for free energy minimization), theoretically, they may also function as semi-separate value signals in terms of respective information gain and preference satisfaction. In this way, convergence of the H/E-S and its ventromedial prefrontal collaborators upon the accumbens core—and thereby nigral motor dopamine (Mannella et al., 2013)—may represent physical manifestations of the dual optimization for intrinsic and extrinsic value prescribed by active inference as a normative account of intelligence. This kind of convergent control based on heterogeneous (fundamental) value signals is notable, as it is becoming increasingly clear the H/E-S is more than just a temporary memory buffer, but rather may constitute a primary basis for autonomous functioning for vertebrates as adaptive cybernetic systems, as highlighted in Figure 6.

## G(Eneralized-)SLAM as Core Cognitive Process

As described above, the H/E-S and its functional relationships with the neocortex may be understood as implementing a kind of Kalman variational autoencoder (Fraccaro et al., 2017). In this capacity, the H/E-S may provide inspiration for developing advanced SLAM architectures. In its dual role as both memory and control system, the H/E-S has been further optimized for facilitating comparisons between largescale patterns (e.g., organismic states), which in machine learning terms may be understood as implementing something akin to energy-based contrastive learning (Marblestone et al., 2016; Richards et al., 2019; Mazzaglia et al., 2022). In this capacity, the H/E-S may provide inspiration for developing architectures capable of engaging in self-supervised learning, counterfactual modeling, and further enabling high-level reasoning abilities including analogical structure mapping (Gentner, 2010; Safron, 2019a), causal inference (Pearl and Mackenzie, 2018), and imaginative planning (Kaplan and Friston, 2018; Safron, 2021b).

As described above, and elsewhere (Safron, 2020b, c, 2021a, b), LatentSLAM’s dual-tier architecture provides an abstract cybernetic interpretation of the H/E-S as the highest (or most integrative) level of heterarchical control for embodied-embedded organisms as they move through physical and imagined worlds in the pursuit of valued goals, so providing a computational/functional account of agency in biological (and perhaps artificial) systems. Further, this hierarchical architecture provides a basis for meta-learning in which slower and more encompassing “outer loop” processes aggregate information over faster “inner loop” processes, so affording the much-desired goal of realizing synergistic inference and generalization of knowledge across experiences (or lessons in curriculums for lifelong learning). Even more, the upper levels of this kind of hybrid architecture may provide a basis for explicit symbolic reasoning (*via* abstract experience graphs) in addition to enactive couplings with the world (*via* adaptive control of poses/views), both of which are likely required for achieving the goal of robust autonomous functioning for artificial systems.

While attempting to navigate towards such destinations may seem excessively ambitious, we would note that work on the extended H/E-S was part of what inspired the formation of some of the world’s leading AI companies such as DeepMind, and continues to be a central part of their research programs (Hassabis and Maguire, 2009; Hassabis et al., 2017; Koster et al., 2018; McNamee et al., 2021). Indeed, it is increasingly being recognized that the spatiotemporal modeling properties of the H/E-S may constitute an invaluable integrative framework for understanding high-level cognition (Whittington et al., 2022). However, we believe a G-SLAM framing might be particularly notable in connecting to the context under which these systems were first selected/shaped by evolution (and development), as well as one of the primary functionalities of the H/E-S that continues throughout the lifespan of organisms. That is, our abilities to navigate both physical and conceptual worlds represent an ongoing challenge for as long as we live. We further suggest the connection between the practical necessities involved in engineering physical systems may provide a particularly valuable source of empirical traction for attempting to specify the roles of particular features of the H/E-S, in that we can draw upon the rich data generated as robots attempt to navigate through the world.

Further, by also drawing upon biological details in designing AI-architectures, we may find ourselves with access to invaluable inductive biases which might be otherwise overlooked. Two examples that come to mind include recent proposals by Bengio and LeCun with respect to “GFlowNets” and “Joint Embedding Predictive Architectures” (Bengio et al., 2022; LeCun, 2022). We believe these efforts in creating autonomous and generally intelligent systems may benefit by incorporating principles of G-SLAM, such as the creation of systems capable of handling loop trajectories as potentially enabling greater open-ended and life-long learning, or in looking towards hybrid systems similar to LatentSLAM as potentially allowing for explicit representations and symbolic processing. While it has often been said that the goal of AI is to create the “cognitive equivalent of an airplane wing,” we would suggest that the magnitude of the challenge may be far greater (more akin to building a fully functioning plane or space ship), and the problems of navigating through under-constrained architectural (and learning curricula) design-spaces may be unsurmountable without biological inspiration/grounding.

While LatentSLAM continues to be refined, we believe these kinds of architectures provide a general framework for understanding core elements of minds and brains. Indeed, to localize something within a spatialized reference frame—which itself is impacted by the entities it maps/graphs— may be what it means to “understand” and “explain” something (Lakoff and Johnson, 1999), and possibly even to experience anything at all (Safron, 2020a, 2021a, b). That is to understand is to be able to adopt a stance (or pose) from which elements and their inter-relations may be mapped (or localized), as if projected onto a plane whereby they are made visible for inspection (or navigation). We believe these etymological considerations on the nature of knowledge may be more than “mere” metaphors but could point to the fundamentally embodied nature of minds.

We not only suggest that all thought may be understood as navigating between representations that are being localized and mapped (or graphed) within an organizing conceptual domain, but all communication may be understood as the transmission of such structures (as trajectories) between minds (Zurn and Bassett, 2020). While the simultaneity of generalized localization and mapping in cognition may not be obvious upon introspection, this is more clearly the case when considering unfamiliar concepts. For such novel domains, relationships between concepts and broader organizing schemas involves the same kind of challenges of circular inference as found in SLAM. That is, when we are first attempting to understand a conceptual domain, we do not know how to effectively connect the entities whose shared features and relations motivate the construction of organizing schemas. However, without such higher-order abstractions and the predictive (or compressive) capacities they provide, it is unclear which features of and relations between entities are relevant for shared structure learning.

Heuristic algorithms may be invaluable in the bootstrapping process, such as the kind of clustering involved in the hierarchical Dirichlet process (Griffiths T. et al., 2019), models of category formation *via* analogical alignment (Kuehne et al., 2000), or concept derivation as abstraction over episodes (Mack et al., 2018). We agree that such accounts may speak to fundamental mental processes, but we also suggest that rather than static feature maps, such nonparametric (Bayesian) structure learning may apply to paths through mapped/graphed domains. This is part of why we emphasized our use of the Fisher distance measure above, as an information metric that naturally applies to trajectories may potentially provide the most valid (and potentially predictive) means of assessing similarity/dissimilarity between entities in feature spaces. Indeed, one of the most notable aspects of thinking is its sequential operation and sensitivity to path dependencies. While abstract conceptualization does allow for a good deal of cognitive flexibility, cognition is still largely defined by deriving knowledge *via* particular “chains of reasoning,” or “paths” through mental space.

Regardless of the particular routes by which we reach the heights of category learning, the formation of such abstract representations constitute what may be the most powerful aspect of our intelligence in terms of generalizable knowledge that can robustly transfer across particular episodes (Marcus, 2020). Such abstract categories further allow for the kinds of structured representations whose importance was emphasized in decades of work in (non-radically-enactive) cognitive science and “good old fashioned AI.” The significance of such knowledge structures may prove even greater in light of the advent of graph networks within the context of geometric deep learning (Battaglia et al., 2018)—and symbolic regression as potentially representing a further degree of abstraction (Cranmer et al., 2020). Such graphical representations are of increasing interest because of both their interpretability as well as their extraordinary efficiency for modeling physical systems. With our models of node duplication and graph-relaxation, LatentSLAM provides a biologically plausible and computationally-tractable account of how such cognitive schemas may be formed and modified through experience. This is notable in that finding principled means of creating and modifying particular structures for graph neural networks (GNNs) remains an ongoing challenge. But if such challenges can be surmounted, then we may achieve the promise of neurosymbolic AI in combining the power of connectionism with reasoning over explicitly represented (and related) symbols (Garcez and Lamb, 2020; Greff et al., 2020). More specifically, we believe that the ability of the H/E-S to create navigable spaces populated by high-level attracting states may also provide a basis for creating *“ad hoc”* (Barsalou, 1983) GNN structures for different purposes.

While the relationships between place cells in the H/E-S (or nodes in LatentSLAM) can be understood as a kind of GNN, we believe it would be more accurate to characterize these models as graph nets, in that they represent relations—or semantic pointers (Blouw et al., 2016)—for hierarchically lower graphs. While these details have yet to be incorporated into LatentSLAM, it has been suggested that heteromodal association cortices may constitute a shared latent space across (autoencoding) cortical hierarchies with quasi-topographic characteristics akin to those found with GNNs (Safron, 2020b, 2021a, b). While the H/E-S has significant interactions with the entire cortical heterarchy, connectivity is most substantial for deeper (or hierarchically higher) portions of cortex, consistent with its potential role as a kind of graph network. The degree to which these machine learning analogies may apply to brain functioning is yet to be determined, but they nonetheless represent a promising direction for creating artificial systems that recapitulate the properties of natural intelligences (Greff et al., 2020).

Intriguingly, the work in which brains were proposed to entail GNN-type computation was developed independently of LatentSLAM. However, similarly to how LatentSLAMs only uses views and proprioceptive poses for specifying particular experiences to be mapped (Figure 2), this other work proposed that sufficient bases for agentic world modeling may involve conjoined visuospatial and somatospatial modalities, potentially (but not necessarily) understood as respective grid and mesh-pose GNNs. In the model of episodic memory and imagination described above (Figure 6), H/E-S trajectories are used to orchestrate state transitions between these experiences as the “stream of consciousness” (James, 1890). While many aspects of cognition are unconscious, “thinking” and “reasoning” are usually considered to involve sequentially generated conscious operations. Notably, the formal conceptualization of computation may have been largely inspired by Turing introspecting his own consciousness in the process of doing mathematics (Dehaene, 2014; Graves et al., 2014). Given that it is unclear that we can be conscious of anything that lacks grounding in somatic modalities and their abilities to change (and be controlled) through time, then all thinking/reasoning may potentially be understood as involving the kinds of action selection and modeling described by LatentSLAM.

Fully describing the potential correspondences between SLAM and high-level cognition is beyond the scope of a single publication (Table 1), but before concluding we will briefly comment on the importance of loop-closures and thresholds for graph-relaxation and node duplication. In brief, we may understand a (generalized) loop-closure event as a primary factor contributing to the feeling of understanding and insight (Gopnik, 1998; Fonken et al., 2020; Oh et al., 2020). After an initial period of relatively ambiguous exploration, the formation of a causal account (or trajectory through a concept space/graph) would allow for a rapid decrease in prediction-error (Joffily and Coricelli, 2013), or increase in compression (Schmidhuber, 2010). While some individuals may be relatively insensitive to these feelings of (potentially sudden) conceptual familiarity (Hou et al., 2013; Ben-Yakov et al., 2014), others may potentially be overly sensitive (e.g., “déjà vu” and other kinds of false positive inferences), with the specific functional tradeoffs involved depending on particular contexts (DeYoung, 2015; Blain et al., 2020; Safron and DeYoung, 2021).

Events in which this kind of cognitive closure is achieved provide special opportunities for updating H/E-S models (or categories) *via* graph-relaxation and node duplication. A variety of relevant parameters can be identified (Figure 3), whether in terms of thresholds for detecting loop-closures, the extent to which graphs may be relaxed, or the ease with which new nodes are created. However, in a G-SLAM context of trying to model cognition more generally, we may think of loop-closure recognition thresholds as sensitivity to cumulative prediction error increases/decreases, graph-relaxation as changing attractor dynamics within the H/E-S and neocortex on multiple scales, and node duplication as the establishment of new local ensembles of effective connectivity (cf. chained bump attractors)—and potentially (but not necessarily) involving neurogenesis, for which it is notable that the hippocampus is one of the few places where this phenomenon is reliably observed. These core SLAM processes may depend on multiple factors, including neuromodulators such as dopamine and serotonin (Safron, 2020c; Safron and Sheikhbahaee, 2021), as well as on Bayesian priors (or “yesterday’s posteriors”).

With respect to the previously described example of differential tuning thresholds for mapping the structure of aisles (Figure 5), we may potentially have a crucial source of individual differences in cognition. In theory, G-SLAM may be pointing to (or localizing) a cognitive spectrum (and potential basis for differential diatheses) spanning autism and schizophrenia (Byars et al., 2014; Crespi and Dinsdale, 2019). Theoretically, we may even expect to see these kinds of variations in SLAM maps in the drawings of autistic and schizophrenic individuals (Morgan et al., 2019; Philippsen and Nagai, 2020).

Speculatively, not only may the conceptual understanding of that which is being drawn be mapped and navigated by the H/E-S as SLAM system, but the eye movements (Wynn et al., 2020) and hand motions involved in skilled actions such as drawing could themselves be orchestrated according to hippocampal trajectories as a basis for chained equilibrium setpoints (Latash, 2010). Even more speculatively, it could even be the case that further degrees of sophisticated control—as inference (Kaplan and Friston, 2018; Friston et al., 2021)—are bootstrapped by simultaneously localizing and mapping the body itself as a kind of space/graph, so allowing for more rarefied and general SLAM capacities over the course of development. In this view, much of cognitive development would involve initial phases of using the H/E-S to learn intentional control over either overtly or covertly expressed motor patterns, which then become automatized (or amortized) by the thalamic-cerebellar system (Safron, 2021a; Shine, 2021) and dorsal striatal-cortical loops (Mannella et al., 2013), so freeing up the G-SLAM system for further high-level predictive modeling and control. Can the body itself be understood as a mapped spatial domain, or is this just a way of speaking without any useful technical correspondences? How far can we go with using these patterns of linguistic use as hypotheses regarding cognitive processes and underlying neural mechanisms? Could it even be the case that the phenomenology of embodiment involves navigation through and mapping of body maps *via* these cross-modal interactions, which when disrupted could potentially contribute to altered states of consciousness or potentially clinical conditions such as depersonalization (Safron, 2020c; Ciaunica and Safron, 2022)?

With respect to personhood, beyond its foundational role for autonomous functioning, widespread orchestration of value-canalized trajectories through biophysical phase space by the H/E-S also enables the development (and ongoing functioning) of the spatiotemporally-extended processes required for autonoetic and autobiographical self-consciousness. In addition to constituting major transitions in evolution, the advent of such self-reflective capacities may have been required for the construction of advanced social coordination and a (shared) symbolic order of being. While such rarefied processes may be well-beyond anything we are close to engendering in (abiotic) machines, it may be the case that we are forced to recapitulate these kinds of H/E-S functionalities if we are to successfully arrive at the destination of creating robustly autonomous and general artificial intelligences.

Indeed, G-SLAM parameters may constitute the most important source of variation we can identify both between and within individuals. To venture deep into unknown speculative territory, the H/E-S may be the source of key adaptations contributing to the evolution of cognitive modernity through (potentially proto-schizotypal) flexibly creative cognition and the birth of cumulative culture, which in time came to represent what may be the “secret of our success” as a species and the greatest of all major transitions in (generalized) evolution (Premack, 1983; Gentner, 2010; Hofstadter and Sander, 2013; Henrich, 2017; Safron, 2019b, 2020c; van den Heuvel et al., 2019; Dehaene et al., 2022). While such models extend far beyond domains of knowledge for which we have well-developed maps, we believe such possibilities are worthy of further exploration.

### Present limitations and future directions for G-SLAM

While we describe experiments for LatentSLAM in other publications (Çatal et al., 2021a), future work should attempt to explicitly illustrate G-SLAM principles with experiments and mathematical models/simulations. Further, while approaches to localization and mapping may be diverse, this does not mean that all technical solutions involved are best described as SLAM problems. However, we believe that analogues of processes like loop closure and node duplication (and pruning) with respect to trajectories through cognitive spaces would constitute strong evidence for the value of a generalized SLAM perspective. It is also important to note that symbolic processing in the brain involves more than the H/E-S. For instance, a substantial amount of symbolic communication is linguistic in a way that could be described in terms of a hierarchical control system for vocal production and hearing (gestural communication could provide another illustrative example). While such action-perception cycles need not involve the H/E-S, we also believe their functioning may potentially be enhanced *via* H/E-S orchestration of high-level dynamics (e.g., channeling neuronal manifolds along particular trajectories).

We also believe it will be valuable to explore research attempting to combine SLAM and various forms of semantic processing in robotics/AI (Kostavelis and Gasteratos, 2015; Sünderhauf et al., 2017; Garg et al., 2020). Not only does such work illustrate the complexity of SLAM problems and how they may (and must) be integrated with other cognitive processes (cf. artificial consciousness?), but it also points to other ways in which robotics can be used to inform our understanding of minds, whether biologically grown and artificially engineered). Finally, while we focus on a particular SLAM architecture developed within the Free Energy Principle and Active Inference framework, we believe it will be fruitful to consider other approaches as well, many of which are extremely well developed and sophisticated in their own right (Penny et al., 2013; Madl et al., 2018; Stoianov et al., 2022; Taniguchi et al., 2022).

## Conclusions

We have searched through broad and diverse terrains in considering the ideas above, covering a lot of ground. To try to come full circle, we have described technical details of a machine learning architecture for autonomous robot navigation, discussed particulars of biological systems for realizing these functionalities in brains, and started to explore how these principles may provide a framework for understanding all high-level cognition in terms of simultaneous localization and mapping in space (broadly construed to include conceptual spaces). We have only begun this journey, but we believe the destination is promising, and we invite others to join us in exploring this framework for understanding the nature of thought. Some might contend that “prediction” or “modeling” are more encompassing and fundamental than a generalized SLAM perspective, and we would not disagree. However, we believe that G-SLAM is unique in allowing for all these perspectives to be combined with the principles of ecological rationality that constituted the primary selective pressures for high-level cognition over the course of evolution and development. We suggest this neuroethological perspective will be invaluable in allowing us to “carve nature at its joints”, in terms of identifying the most important features of functioning for the hippocampal/entorhinal system and its connections to the rest of the brain [and body (and world)]. We further believe that G-SLAM is unique in the extent to which it connects to nature(s) of experience, where we do in fact exist in a spatial world through which we must navigate, and where it is difficult to find aspects of mind not impacted by this fundamental physical situatedness. In light of these sources of potential insight, we believe that G-SLAM represents the way forward for understanding complex minds, and potentially for building them, if we can find sustainable paths into the unexplored territory of the future.

## Data Availability Statement

The original contributions presented in the study are included in the article, further inquiries can be directed to the corresponding author.

## Author Contributions

AS, OÇ, and TV conceptualized G-SLAM and the main ideas for this manuscript. OÇ and TV conceived and performed the LatentSLAM experiments. AS contributed his knowledge gained from his ongoing study of the hippocampal/entorhinal system literature. All authors contributed to the article and approved the submitted version.

## Funding

OÇ is funded by a Ph.D. grant of the Flanders Research Foundation (FWO). This research received funding from the “AI Flanders” program of the Flemish government.

## Figures and Tables

**Figure A1:**
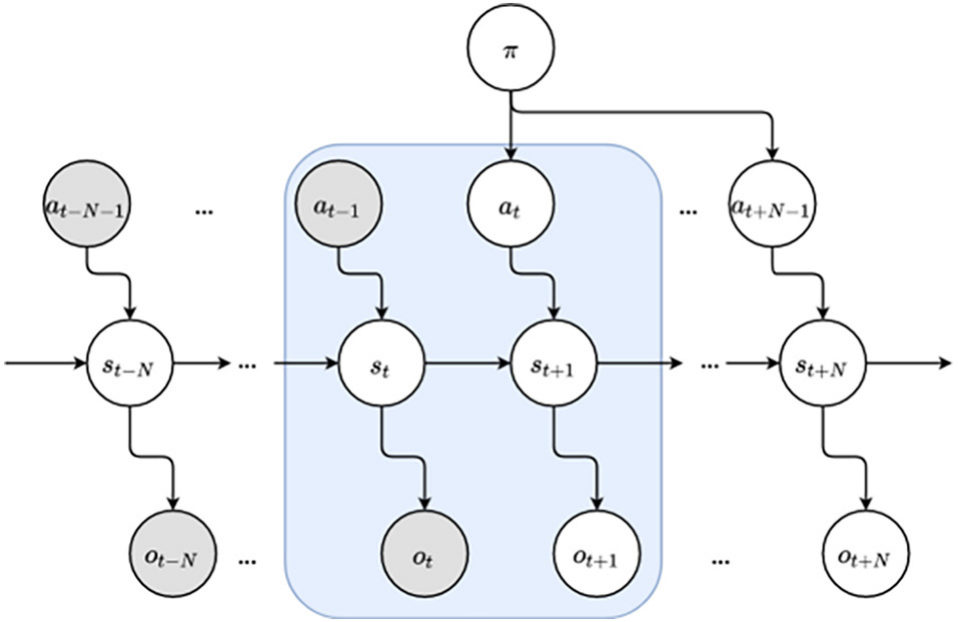
Graphical representation of a generic active inference model. Gray nodes indicate that the corresponding value is observed, white nodes need to be inferred.

**Figure A2:**
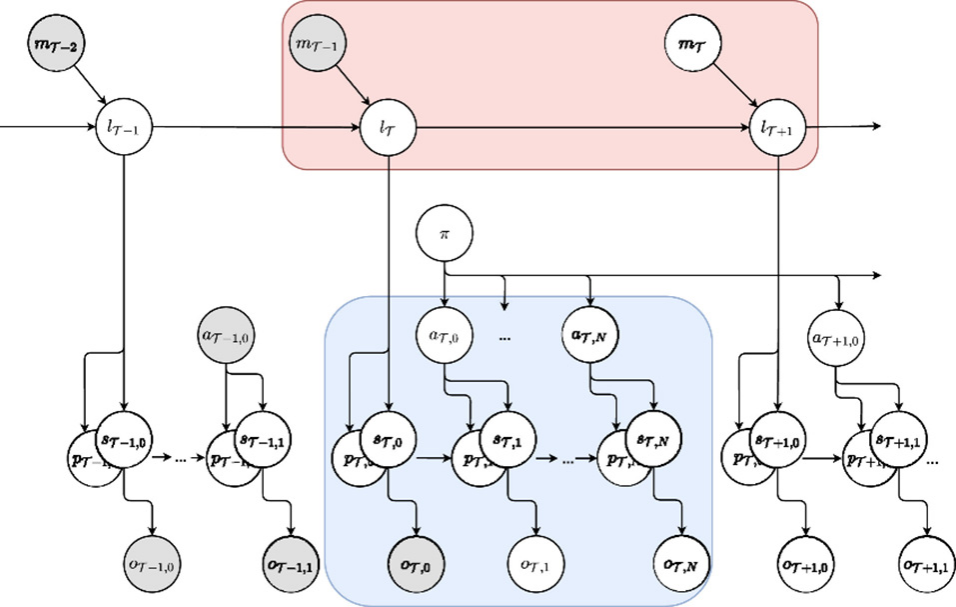
Graphical overview of the hierarchical navigation model.

## Appendix A: LatentSLAM Mathematical Model

The latentSLAM model for SLAM can be seen as a two-level active inference hierarchy working together to enable navigation. The lower-level abstracts actions and observations from the physical world into an abstract representation. The higher-level takes the lower-level abstractions as inputs and creates a global abstraction over them. In this appendix we will go into the mathematical details of these models.

### Generic active inference model

Both levels of the hierarchy form an instantiation of an active inference model (Figure A1). This means that each level forms a generative model over its own actions and observations. We assume the environment is modeled up to a certain time horizon T by the agent as a POMDP with joint probability distribution

$$P\text{(}\tilde{o}\text{, }\tilde{s}\text{, }\tilde{a}\text{, }\pi \text{) = }P(s_{o})P(\pi )\prod_{t\;=\;1}^{T}P(o_{t}|s_{t})\;P(s_{t}|s_{t-1},a_{t-1})\;P(a_{t-1}|\pi )$$

Where tildes indicate sequences of the corresponding variables, *a* indicates the action, *o* the observation, *s* the latent states and *π* the policy.

The agent needs to infer the posterior belief on latent states $p(\tilde{o},\tilde{s},\tilde{a})$. In order to achieve this, we use a variational approximation of the true posterior, which we parametrize as

$$Q(\tilde{s}|\tilde{o},\;\tilde{a})=Q(s_{0}|o_{0})\prod_{t\;=\;1}^{T}Q(s_{t}|s_{t-1},\;a_{t-1},\;o_{t})$$

Note that in all following discussions we will use *Q* to designate a (variational) posterior and *P* as a prior distribution. As we the agent is acting according to the free energy principle, it is actively minimizing its variational free energy. Which we posit here as

$$\text{F = }D_{KL}[Q(\tilde{s}|\tilde{o},\;\tilde{a})||P(\tilde{s},\;\tilde{a})]-{𝔼}_{Q(\tilde{s}|\tilde{o},\;\tilde{a})}[\log\;(P(\tilde{o}|\tilde{s})]$$

For a more detailed description of the derivation of *F*, we refer the reader to. The generative model and the free energy form only one aspect of active inference. The agent not only needs to infer states from the present, but also actions for the future. This is achieved through the expected free energy, which we define for a future timestep *τ* and a given policy *π* as

$$G\left(\text{π},\text{τ}\right)=D_{KL}[Q(s_{\tau }|\;\text{π})||P\left(s_{\tau }\right)]+{𝔼}_{Q\left(s_{\tau }\right)}[H(P(o_{\tau }|s_{\tau }))]$$

Summing over the future timesteps then gives the expected free energy for a given policy.

### Navigation model

Similar to the generic active inference model, we start by defining the generative model in terms of a joint distribution over its parts.

$$P\left(\tilde{o,}\tilde{s,}\tilde{a},\tilde{l},\tilde{p},\tilde{m},\pi \right)=P(\tilde{o},\;{\tilde{s}}_{i>0},\tilde{a},\;{\tilde{p}}_{i>0},\pi \;|\;\tilde{l},{\tilde{s}}_{0},{\tilde{p}}_{0})P\left(\tilde{l},\tilde{m},\;{\tilde{s}}_{0},{\tilde{p}}_{0}\right)$$

Where *o, s*, and *π* keep their earlier definition and we now introduce the pose *p*, location *l* and move *m* to the discussion. This joint distribution naturally decomposes into two new joints over a subset of variables, allowing the independent treatment of higher-level and lower-level navigation. The resulting graphical model is shown in Figure A2.

If we look at the free energy of this model, we get

$$F_{hierarchical}={𝔼}_{Q}\left[\log Q\left(\tilde{s},\tilde{p}\right)-\log P\left(\tilde{o},{\tilde{s}}_{i>0},\tilde{a},{\tilde{p}}_{i>0}\text{|}\tilde{l},{\tilde{s}}_{0},{\tilde{p}}_{o}\right)+\log Q\left(\tilde{l}\right)-\log P\left(\tilde{l},\tilde{m},{\tilde{s}}_{0},{\tilde{p}}_{0}\right)\right]=\;F_{low}+F_{high}$$

Allowing for a clean separation in the creation of the lower and higher-level state models.

#### Lower-level model

Using the same approximations for the lower-level model as in the generic active inference model, we write the free energy as

$$F_{low}=\sum_{t}{𝔼}_{Q}[\log Q(p_{t})-\log P(p_{t}|p_{t-1},a_{t-1},s_{t})]+D_{KL}[Q(s_{t})||P(s_{t}|s_{t-1},a_{t-1})]+{𝔼}_{Q}[-\log P(o_{t}|s_{t})]$$

From this we recover the same generative model for the observations and observational latent states as before in the generic case, however, the model is now supplemented with a term responsible for the pose estimation aspects. In effect, this means that we do not use any pose information for the visual perception part of the model. Note that the pose estimation is in fact conditioned on the current perceptual state estimate.

As might be expected from the free energy formulation, the lower-level perception is implemented as a generic active inference model. The pose estimation aspect is deliberately left as an expected difference between the pose posterior and prior.

The pose prior is implemented as simple dynamics model from the action velocities used to control the agent, i.e.,

$$\begin{array}{c} \theta_{t}=\theta_{t-1}+\upsilon_{a}\Delta_{t}\\ x_{t}=x_{t-1}+\upsilon_{l}\Delta_{t}\\ y_{t}=y_{t-1}+\upsilon_{l}\Delta_{t} \end{array}$$

With *v_a_*and *v_l_* the angular and linear velocity of the agent and *x,y,θ* the coordinates and rotation in the plane of the agent.

The pose posterior *Q (p_t_)* is implemented as a CAN with energy dynamics described as

$$\varepsilon_{\Delta x,\Delta y,\Delta \theta }=\text{exp}\frac{-\Delta x^{2}-\Delta y^{2}}{k_{p}^{exc}}\exp\frac{-\Delta \theta^{2}}{k_{d}^{exc}}-\exp\frac{-\Delta x^{2}-\Delta y^{2}}{k_{d}^{inh}}\exp\frac{-\Delta \theta^{2}}{k_{d}^{inh}}$$

With *k_d_* and *k_p_* the variance constants for place and direction, and the superscript *exc* and* inh* used to indicate whether the effect is inhibitory or excitatory. The resulting behavior is locally excitatory and globally inhibitory. The conditioning on observatory state *s_t_* is achieved by creating an extra excitatory link with a state-pose episodic memory.

#### Higher-level model

Starting again from the free energy functional

$$F_{high}=\sum_{𝒯}D_{KL}[Q(l_{𝒯})||P(l_{𝒯}|l_{𝒯-1},m_{𝒯-1})]+{𝔼}_{Q}[-\log P(s_{𝒯,0}|l_{𝒯})-\log P(p_{𝒯,0}|l_{𝒯})]$$

We again see the classical active inference model emerging. Note the usage of 𝒯 instead of *t* to indicate that this model operates on a different timescale. Likewise, only the initial lower-level states for that inference cycle appear in the likelihood (remember that this model uses the states of the lower-level as observations). Here again, the actual implementation of these models is geared towards a navigational task. In order to infer the location, the prior distribution of locations is implemented as an experience graph. Each node in the graph incorporates a state, pose pair to link it with the lower level. Links between the nodes indicate a connection traversable on the lower level. The dynamics model $P(l_{𝒯}|l_{𝒯-1},m_{𝒯}-1)$ is deduced from the adjacency matrix of the graph. The posterior belief $Q(l_{𝒯}|s_{𝒯,t},p_{𝒯,t})$ is build by assigning probability inversely proportional to the cosine similarity and Euclidean distance between current state pose pair and the pairs reachable from the current node.

Map updates also trigger a graph-relaxation pass, in order to facilitate loop-closures. The graph-relaxation phase shifts the stored poses in each experience map node according to

$$\Delta p^{i}=\frac{1}{2}\left[\sum_{j=1}^{inbound}(p^{j}-p^{i}-\Delta p^{ij})+\sum_{k=1}^{outbound}\left(p^{k}-p^{i}-\Delta p^{ij}\right)\right]$$

#### Action inference

So far, we have only discussed the state inference aspects of the navigation model, however, action inference is also an important aspect of active inference. The expected free energy for the lower-level model is

$$G_{low}(\pi ,r)=D_{KL}[Q(s_{\tau },p_{\tau }|\pi )||Q(s_{T+1},p_{T+1}|l_{T},m_{T})]+{𝔼}_{Q(s_{T})}[H(P(o_{\tau }|s_{\tau }))]$$

The prior preferences in this equation are provided by the higher-level model, and form targets to achieve within the single timestep of that level. High level targets are then extracted according to

$$G_{high}(\pi ,r)=D_{KL}[Q(l_{\tau }|\pi )||P(l_{\tau })]+E_{Q(l_{T})}[H(P(p_{\tau ,0}|l_{\tau }))+H(P(s_{\tau ,0}|l_{\tau }))]$$

Action selection happens than according to a two-phase planning process. First, at the coarser higher-level, second at the fine-grained lower level. This allows for a reduction in search space for a given trajectory.
